# Leaf Epidermis of the Rheophyte *Dyckia brevifolia* Baker (Bromeliaceae)

**DOI:** 10.1155/2013/307593

**Published:** 2013-06-24

**Authors:** Ghislaine Maria Lobo, Thaysi Ventura de Souza, Caroline Heinig Voltolini, Ademir Reis, Marisa Santos

**Affiliations:** ^1^Universidade Federal de Santa Catarina, Centro de Ciências Biológicas, Departamento de Botânica, 88040-900 Florianópolis, SC, Brazil; ^2^Universidade Federal da Fronteira Sul, 88750-000 Realeza, PR, Brazil; ^3^Herbário Barbosa Rodrigues, 88301-302 Itajaí, SC, Brazil

## Abstract

Some species of *Dyckia* Schult. f., including *Dyckia brevifolia* Baker, are rheophytes that live in the fast-moving water currents of streams and rivers which are subject to frequent flooding, but also period of low water. This study aimed to analyze the leaf epidermis of *D. brevifolia* in the context of epidermal adaptation to this aquatic plant's rheophytic habitat. The epidermis is uniseriate, and the cuticle is thickened. The inner periclinal and anticlinal walls of the epidermal cells are thickened and lignified. Stomata are tetracytic, located in the depressions in relation to the surrounding epidermal cells, and covered by peltate trichomes. While the epidermal characteristics of *D. brevifolia* are similar to those of Bromeliaceae species, this species has made particular adaptations of leaf epidermis in response to its rheophytic environment.

## 1. Introduction

The family Bromeliaceae comprises about 54 genera and approximately 3000 neotropical species [1] which are distributed in Brazil in a wide variety of environments [2, 3]. The species are usually grouped into three subfamilies: Pitcairnioideae, Tillandsioideae, and Bromelioideae [3]. Pitcairnioideae is the most primitive subfamily, being comprised of terrestrial and *saxicola* species characterized by the absence of absorptive trichomes and storage tanks [4]. The genus *Dyckia* belongs to this subfamily and is represented by twelve species in the State of Santa Catarina, Brazil, with some endemic species along several river valleys [2].

*Dyckia brevifolia* Baker is a rheophyte species endemic to islands or rocky banks along the beds of swift-running streams and rivers in the Atlantic slope of Santa Catarina State, Brazil, with wide and significant, but discontinuous, distribution [2, 5, 6]. According to Van Steenis [7, 8], the biological group called rheophytes consists of plants that occur exclusively on the banks of swift-running streams and rivers that experience frequent and sudden floods, but also periods of low water. Such environments are often targeted for construction of dams to capture hydroelectric power, and, as a consequence, these plants are at risk of extinction. According to Van Steenis [7, 8], rheophytes show similar adaptations and specializations associated with the habitat where they occur. In particular, *Dyckia brevifolia* is a heliophyte able to tolerate full sunlight, but it is also a species that can adapt to the extremes of river flow, either by submergence during floods or dehydration during periods of low tide [2, 5, 6].

The adaptations that enable a plant to survive in a particular environment originate from continuous selective pressures exerted by that environment and can be manifested morphologically and anatomically [9–11]. The epidermis, which is in direct contact with the environment, can present striking structural changes in response to environmental factors [12].

The leaf epidermis in Bromeliaceae is composed of a single layer of cells, rarely with papillose, thin cuticle; peltate trichomes, consisting of peduncle and distal large shield; and stomata, usually covered by trichomes [13]. Silica bodies have been reported as present in the epidermal cells of several species of Bromeliaceae [13–16]. According to Prychid et al. [17], this characteristic accumulation of silica in Bromeliaceae is similar to what happens in other families of monocots. The epidermal cells play an important role in protecting the underlying tissues against dehydration, excessive irradiation, heat loss, and night mechanical injury [18]. The cuticle protects the leaves from excessive radiation, enabling the appropriate intensity of irradiation in chlorenchyma, favoring the activation of chloroplasts [19, 20], and also reducing water loss [21]. The cutin and waxes act as barriers against fungi, bacteria, and insects, and, in more severe environmental conditions, the wax acts as barrier to water loss from the surface of the plant, reducing the wettability of leaves, when cutin is not sufficient [22, 23]. In Bromeliaceae, variation in epidermal structures, especially trichomes and stomata, [14], is related to water economy [13]. Stomata are related to important physiological processes of the plant, such as local exchange of oxygen and carbon dioxide for photosynthesis and respiration and even local diffusion of water vapor transpiration [12, 24]. In hydrophytes, stomata tend to be elevated [25], whereas in xerophytes, they tend to sink below the surface of the epidermis, as a way to reduce sweating during stomatal opening [10]. 

This study describes the morphoanatomic characteristics of the leaf epidermis of *Dyckia brevifolia* Baker, an endemic rheophyte that occurs on the banks of the Itajai-Acu River, in order to increase knowledge about the anatomy of Bromeliaceae and describe the adaptations which enable the survival of this species in its unique rheophytic environment.

## 2. Materials and Methods

The study was made with fully expanded leaves taken from the middle portion of the rosettes of six specimens of *Dyckia brevifolia* Baker collected on the banks of the Itajai-Acu River, one of the three main tributaries of the Itajai River Basin, which is located between the geographical coordinates of 26°55′S–49°07′W and 27°02′S–49°37′W, where the river is subjected to frequent alternating periods of low and high water. The herbarium specimens were deposited in the Herbarium FLOR 36,870. The rosettes of *D. brevifolia*, fixed on rocks, were exposed to intense solar radiation during periods of ebb, but submersion in water during flooding. The climate of the Itajai River Basin is subtropical humid, as defined by Köeppen, being influenced by the existence of high mountains to the west and south which, during the winter, protect plants from the westerly winds and, during the summer, act to raise the temperature. The average annual temperature is 20.1°C, and the average annual rainfall is 1596.2 mm, with almost uniform distribution of rainfall for each month and average annual rainfall of 152.4 days [26].

Samples of *in vivo* and fixed leaves removed from the middle region of blades and sheaths were processed for micromorphological and anatomical studies, as described below.

### 2.1. Optical Microscopy (OM)

Samples were fixed in 2.5% glutaraldehyde buffered with sodium phosphate 0.1 M pH 7.2 and then dehydrated in an ethanol series [27]. Part of the samples was infiltrated in hydroxyethyl methacrylate (Jung's Historesi-Leica), as recommended by the manufacturer, and sectioned with a rotatory microtome. Paradermic and cross sections were stained with Astra Blue and basic fuchsin [28] or with toluidine blue [29]. Another part was infiltrated in paraffin according to Johansen [30] and stained with Astra Blue and basic fuchsin and then acidified with picric acid, as in Luque et al. [31]. The observations and capture of images were made with a Sony P92 digital camera and an optical microscope (OM) (Leica MPS 30 DMLS).

Sections of fresh material were obtained freehand and subjected to reactive Steinmetez [32] to confirm the presence of suberin, lignin, cellulose, mucilage, starch, and phenolics; acidified phloroglucinol [32] to show lignin; thionine [33] for mucilage; and hay and clove oil [30] to confirm the presence of silica. The sections were mounted on semipermanent slides with glycerinated gelatin [33].

### 2.2. Scanning Electron Microscopy (SEM)

Samples were fixed, dehydrated, and immersed in hexamethyldisilazane (HMDS) as a way to substitute CO_2_ critical point by the process of sublimation that reduces surface tension, preventing the collapse of structures [34]. The samples were adhered to aluminum brackets and subsequently metallized with gold to form a layer 20 nm in thickness. The documentation of the material was made by SEM (Phillips XL30).

## 3. Results and Discussion

**Figure 1:**
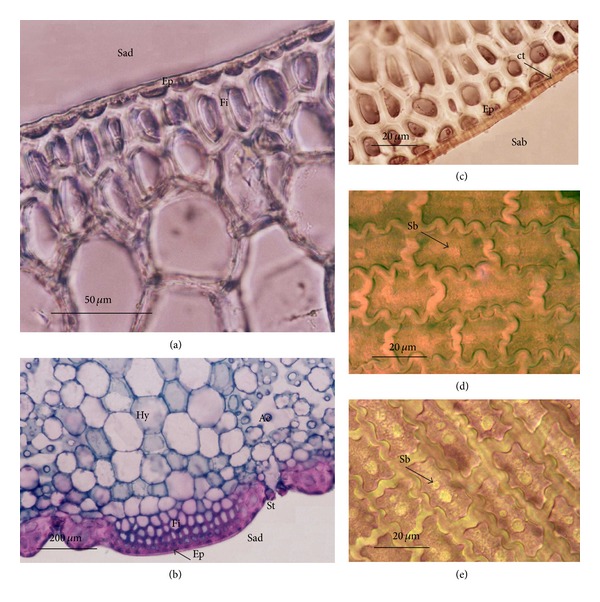
Leaf blade of *Dyckia brevifolia* Baker, as observed in light microscopy. ((a), (b), and (c)) Cross sections. ((d)–(e)) Epidermis frontal view. (a) Flat adaxial surface. (b) Abaxial surface with bumps and depressions. (c) Adaxial surface. (d) Abaxial surface. (e) Abaxial surface, showing the epidermis and subepidermal sclerified cells (fibers) in the coastal zone. (Ae = aerenchyma; Cl = chlorenchyma; Sc = siliceous body; Ct = cuticle; Ep = epidermis; St = stomata; Sab = abaxial surface; Sad = adaxial surface; Fi = fibers; Hy = hydrenchyma.)

In cross section, the leaf blades of *Dyckia brevifolia* have a nearly flat adaxial surface (Figure 1(a)), while the abaxial surface shows protrusions in the coastal areas and depressions in the intercoastal zones (Figure 1(b)). The epidermis is uniseriate (Figure 1(a)), presenting cells with anticlinal walls and inner periclinal walls thickened and lignified (Figures 1(a) and 1(b)), while the external periclinal wall is thin and coated by a thick cuticle (Figure 1(c)) on both faces. According to Haberlandt [18], the wall thickening of the epidermis helps to protect underlying tissues from desiccation, mechanical injury, excessive light by radiation, and night heat loss. In some Bromeliaceae, these functions are assumed by the internal anticlinal wall. Gunning and Steer [23] suggest that the presence of cutin, suberin, and waxes, which coat the plants externally, protect against the action of pathogens, and limit water loss. The presence of anticlinal traces, which must correspond to micropores, is observed in the cuticle (Figure 1(c)). Lyshede [35] notes that cuticular microchannels come from the pectic layer of the cell wall based on red ruthenium staining, further commenting that the birefringence found in these microchannels suggests wax transport. In the cutinized walls, Lyshede [36] assumes that pectin channels can facilitate the passage of water from the surface into the cell. Lyshede [35] also comments that most modern studies have also suggested microchannels as evidence of cutin and wax transport.

**Figure 2:**
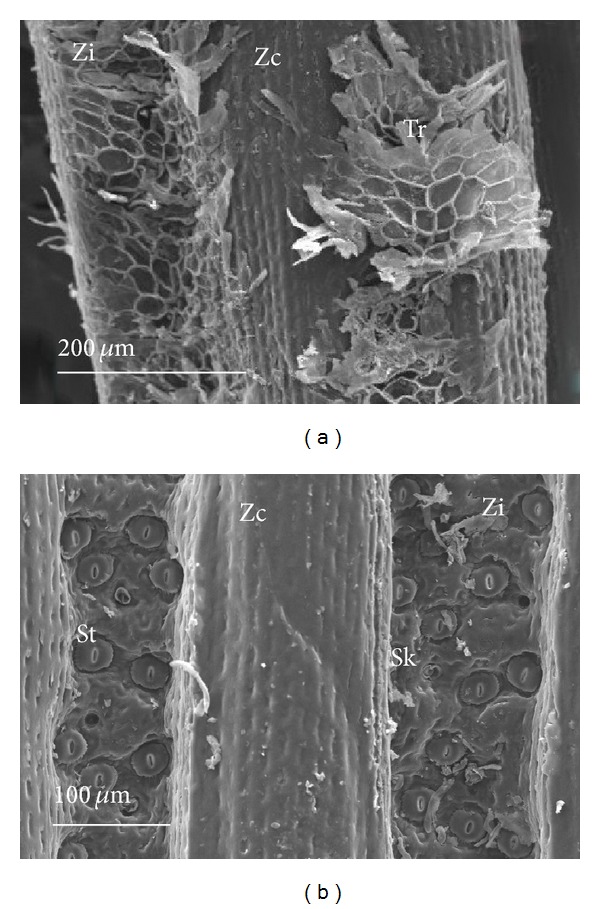
Abaxial surface frontal view of the leaf blade of *Dyckia brevifolia* Baker, as observed in scanning electron microscopy. (a) Peltate trichomes (Tr) covering the intercoastal zones (Zi). (b) Stomata (St) in the intercoastal zones (Zi). (Sk = stalk; Zc = coastal zone.)

**Figure 3:**
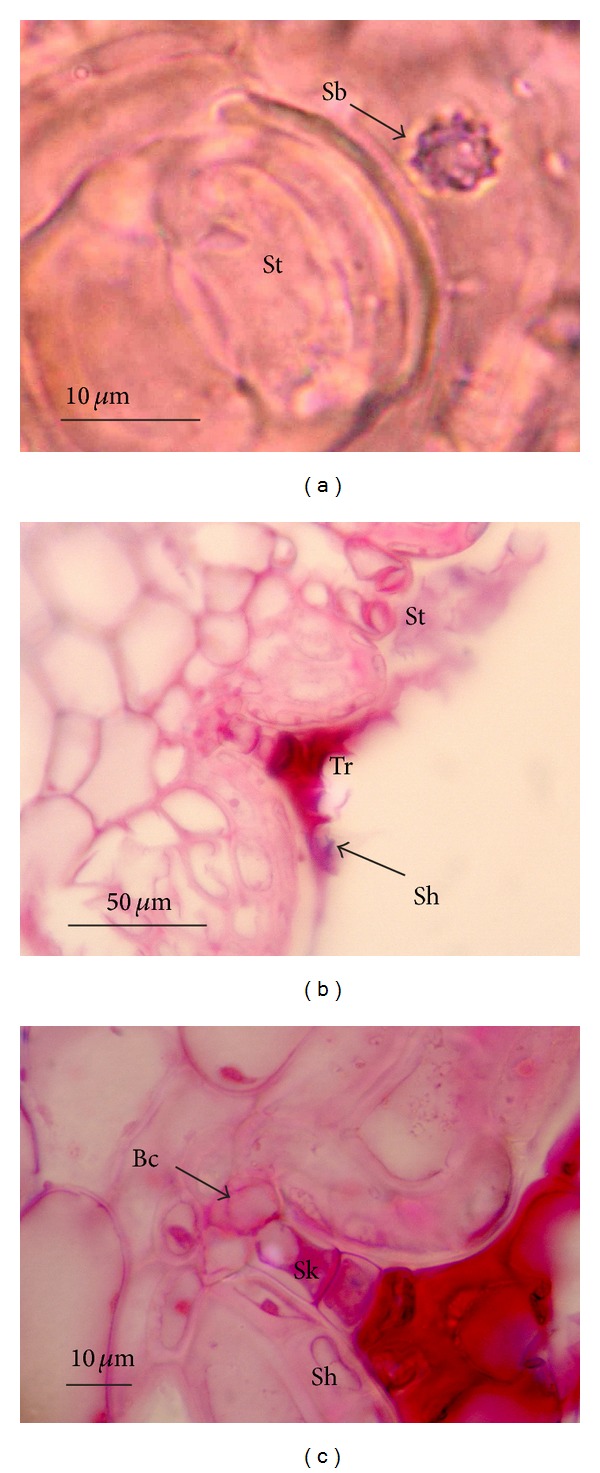
Leaf blade of *Dyckia brevifolia* Baker, as observed in light microscopy. (a) Frontal view of the abaxial surface, showing siliceous body (Sb) with rough surface. ((b) and (c)) Cross section showing abaxial surface. (b) General view showing trichome (Tr) in epidermis depression and stomata (St). (c) Detail showing the trichome formed by basal cells (Bc), stalk (Sk), and shield (Sh).

In frontal view, the leaf blades were observed to have more homogeneous epidermis on the adaxial surface, consisting of ordinary cells (Figure 1(d)), while the abaxial coastal zones exhibited only common cells (Figures 1(d), 2(a), and 2(b)) and intercoastal zones had trichomes (Figure 2(a)) and stomata (Figure 2(b)). The leaf sheaths are devoid of stomata and trichomes, as well as scars of scales, on both surfaces. The common epidermal cells are characterized by having a regular form and sinuous anticlinal walls (Figures 1(d) and 1(e)), in addition to silica bodies consisting of tiny crystals which impart an irregular appearance to the surface (Figures 1(d), 1(e), and 3(a)). Among monocots, Prychid et al. [17] reported that the most common type of siliceous body is spherical with rough and prickly surface. They also observed that only one silica body per cell typically occurs and that the cells containing silica are most commonly found in the epidermis. In Bromeliaceae, the presence of silica bodies, each positioned in the center of adaxial epidermal cell surface, promotes the dispersion of solar irradiation heating, thus reducing the likelihood of photodamage to chlorenchyma [1].

Tomlinson [13] describes the trichomes of Bromeliaceae as complex multicellular structures, consisting of a shell of dead cells and a stalk of live cells located in concavities of the epidermis. The trichomes of *D. brevifolia* are the peltate type (Figures 2(a) and 3(b)). They are located in depressions of the epidermis, and they are composed of base, stalk, and shield (Figure 3(b)). The base is formed by a central cell and four lateral cells. Krauss [14], studying *Ananas comosus*, considers these four lateral cells as subsidiaries to the trichome. This idea is based on the early stages of development when the initial cell of the trichome is expanding and the surrounding cells are multiplying more rapidly, thereby confining the base of the initial cell to the bottom of a depression. In *D. brevifolia*, the stalk, which consists of about three cells with serial disposition, protrudes from the central cell of the base of the trichome (Figure 3(c)). In the distal portion of the stalk, the shield expands perpendicularly (Figure 3(b)) and is formed by cells, variable in number and size, arranged haphazardly, giving an asymmetrical appearance (Figure 2(a)). Proença and Sajo [16] attributed this asymmetrical arrangement of the shield to the unequal length of the wing cells, a characteristic in *D. tuberosa*. Benzing [37] further reports this disordered arrangement of the shield cells as a characteristic of the subfamilies Pitcairnioideae and Bromelioideae. Pittendrigh [4] suggests that leaves in Pitcairnioideae species may show little or no absorption capacity. Meanwhile, Benzing et al. [38] demonstrated absorption capacity of the trichomes of Tillandsioideae, but they also reported the absence of this capability in trichomes of Bromelioideae and Pitcairnioideae, attributing this to the presence of a system of well-developed absorption roots in these two subfamilies. Eames and MacDaniels [9] reported that trichomes may be related to various functions, to a greater or lesser degree, but probably the most important one is the reduction of transpiration resulting from the formation of an additional coverage. The peltate trichomes positioned above the stomata and often located in pronounced intercoastal grooves help to conserve water in many Bromeliaceae [1]. The presence of peltate trichomes, which cover the stomata in intercoastal zones in *D. brevifolia*, for example, should facilitate the formation of an intermediary microclimate between the inside and outside air circulation. The air layer near the leaf surface, a boundary layer, provides a barrier against the loss of water vapor through stomata [39]. 

**Figure 4:**
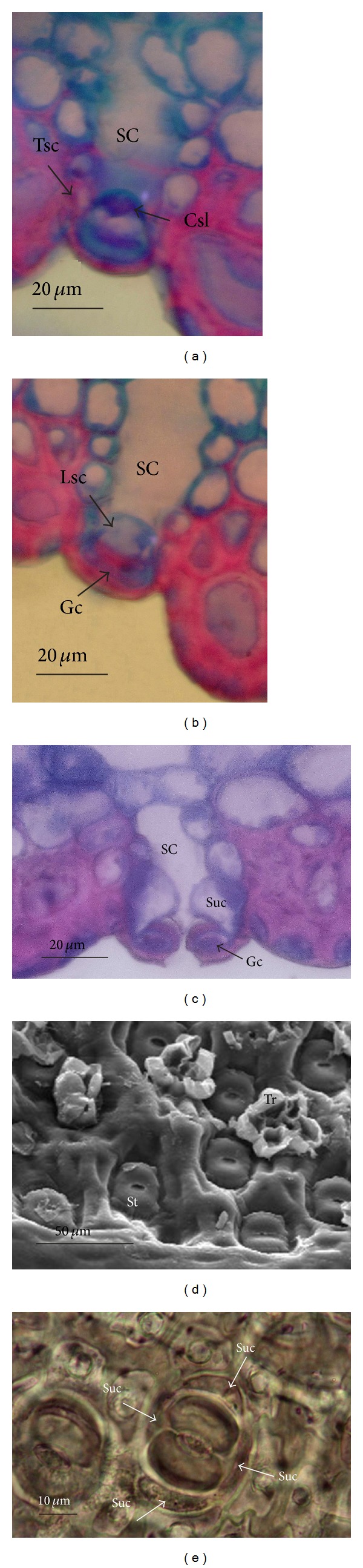
Stomata in the leaf blade of *Dyckia brevifolia* Baker. ((a) and (b)) Transverse-longitudinal sections with detail of the stomatal structure in individual epidermis depressions between sclereid groups, as shown in light microscopy. (c) Cross section in light microscopy, which shows guard cells (Gc), subsidiary cells (Suc), and substomatal chamber (SC). (d) Stomata frontal view (St) depth in the epidermis and trichomes (Tr), showing part of the shield, as observed in scanning electron microscopy. (e) Frontal view of the subsidiary cells (Sc) of the stomata, as observed in light microscopy. (Lsc = lateral subsidiary cell; Tsc = terminal subsidiary cell).

In frontal view, the stomata of *D. brevifolia* are distributed in the longitudinal grooves in areas corresponding to the intercoastal zones of the abaxial surface (Figures 2(b) and 4(a)), being completely covered by peltate trichomes (Figure 2(a)). Furthermore, each stoma is in a single depression, keeping the guard cells in a higher position in relation to the immediately surrounding cells (Figures 4(a), 4(b), and 4(c)).

Determination of stomata type, usually based on observations made in frontal view, cannot be performed in *D. brevifolia*. To explain this, the frontal view of this type of leaf shows the presence of guard cells bordered by two lateral subsidiary cells (Figure 4(d)). Since the first of the two lateral subsidiary cells is obscured by the second, only cross sections can reveal stomata type (Figure 4(c)). Thus, in the present study, the stomata of *D. brevifolia* were determined to be paracytic, as mentioned by Tomlinson [13] for the Bromeliaceae. However, two other subsidiary cells cannot be detected in the frontal view, as they are completely covered up by guard cells. In transverse-longitudinal serial sections of the stomata (Figures 4(a) and 4(b)), two lateral cells can be recognized and two polar cells (terminals) are adjacent to the guard cells. Krauss [14], studying the ontogeny of *Ananas comosus* (L.) Merr. (Bromeliaceae) leaves, reports that the stomata are formed by four subsidiary cells, two lateral and two terminal. Since the stomata of *D. brevifolia* have four subsidiary cells, they are considered tetracytic.

Several epidermal characteristics observed in *D. brevifolia* contribute to the prevention from tissue dehydration during low-water periods; for example, when the plant is under intense solar irradiation we have the following: presence of a thick cuticle, silica bodies and location of the stoma in abaxial surface arranged in longitudinal intercoastal grooves completely protected by trichomes. On the other hand, the thick cuticle associated with stomatal complex structure, which is grooved and protected by peltate trichomes, acts as waterproofing for the leaf structure during periods of flooding and submersion. Thus, the morphoanatomical aspects of *D. brevifolia *are similar to those of other Bromeliaceae species; however, their xeromorphic and hydromorphic characteristics constitute important adaptations to the periods of both low and high water in the rheophytic environment, making their survival possible under these unique environmental conditions.
